# Evaluation of FRET X for single-molecule protein fingerprinting

**DOI:** 10.1016/j.isci.2021.103239

**Published:** 2021-10-05

**Authors:** Carlos Victor de Lannoy, Mike Filius, Raman van Wee, Chirlmin Joo, Dick de Ridder

**Affiliations:** 1 Bioinformatics Group, Wageningen University, Droevendaalsesteeg 1, 6708PB Wageningen, the Netherlands; 2 Department of BioNanoScience, Kavli Institute of Nanoscience, Delft University of Technology, van der Maasweg 9, 2629HZ Delft, the Netherlands

**Keywords:** Structural biology, Biophysics, Mathematical biosciences, Proteomics

## Abstract

Single-molecule protein identification is an unrealized concept with potentially ground-breaking applications in biological research. We propose a method called FRET X (Förster Resonance Energy Transfer via DNA eXchange) fingerprinting, in which the FRET efficiency is read out between exchangeable dyes on protein-bound DNA docking strands and accumulated FRET efficiencies constitute the fingerprint for a protein. To evaluate the feasibility of this approach, we simulated fingerprints for hundreds of proteins using a coarse-grained lattice model and experimentally demonstrated FRET X fingerprinting on model peptides. Measured fingerprints are in agreement with our simulations, corroborating the validity of our modeling approach. In a simulated complex mixture of >300 human proteins of which only cysteines, lysines, and arginines were labeled, a support vector machine was able to identify constituents with 95% accuracy. We anticipate that our FRET X fingerprinting approach will form the basis of an analysis tool for targeted proteomics.

## Highlights

• We propose a FRET-based single-molecule protein identification method
• Peptides are experimentally distinguishable by their fingerprints
• Our approach can classify the constituents of complex samples with 95% accuracy

## Graphical abstract

**Figure:**
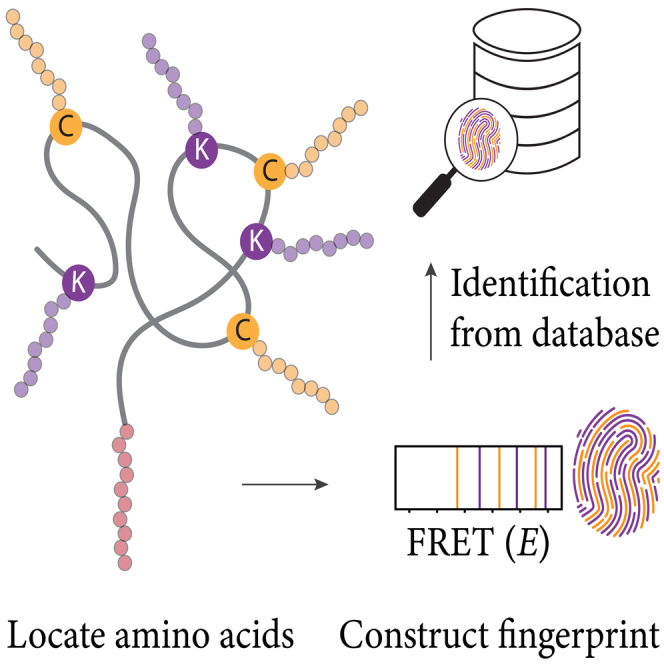

## Introduction

Proteins come in a wide variety of shapes, sizes, and forms. Each is attuned to fulfill one or more of the many functions that are essential to living cells, including the catalysis of metabolic reactions, replication of genetic information, provision of structural support, and transport of molecules. To fully understand the biological processes taking place in a cell, it is critical to identify and quantify constituents of its proteome at any given time during the cell cycle.

Mass spectrometry (MS) is currently the gold standard for protein identification and quantification. Over the past decades, MS techniques have improved tremendously in terms of accuracy and dynamic range; however, detecting and distinguishing all proteins in complex samples remains challenging. Many biologically and clinically relevant proteins such as signaling molecules and disease biomarkers occur in such low abundance that they remain undetectable by MS (Zubarev 2013). Moreover, the proteome complexity increases through alternative splicing or posttranslational modifications, as a single gene can produce dozens of distinct protein varieties, referred to as proteoforms (Aebersold et al. 2018). Not all of these proteoforms can be distinguished by current approaches. As such, there is considerable incentive for the development of new protein sequencing methods that operate at the single-molecule level (Restrepo-Pérez et al., 2018; Alfaro et al., 2021).

Single-molecule techniques have boosted DNA sequencing, allowing for the identification of individual nucleic acid molecules, and are now routinely used for genome and transcriptome mapping of single cells (Gawad et al., 2016). However, the search for single-molecule protein sequencing techniques is not trivial owing to the high complexity of protein molecules compared with DNA molecules. For example, the DNA code consists of only four nucleotides, whereas there are twenty different amino acids for proteins. Furthermore, low abundant DNA molecules can be enzymatically amplified outside the cell, whereas such an enzyme is absent for proteins.

Novel single-molecule protein analysis methods have been proposed to circumvent this additional complexity. Only a subset of the theoretically possible combinations of polypeptide chains occurs in nature, and a fraction of that subset is of importance in a given research setting. Therefore, proteins may be identified by reading out a signature of incomplete information, which is then compared with a database of relevant signatures. We refer to this approach as protein fingerprinting, and to said protein signatures as protein fingerprints. It has been shown that sufficiently distinct protein fingerprints only require the readout of a small subset of residue types (Swaminathan et al., 2015; Yao et al., 2015; Ohayon et al., 2019). In particular, simulations indicated that the majority of human proteins were uniquely identifiable if cysteine and lysine residues were orthogonally labeled and read out sequentially (Yao et al., 2015).

Several novel protein fingerprinting methods based on the readout of a subset of residue types have recently been demonstrated, most of which require linearization of the polypeptide chain to allow for the determination of the residue order (Nivala et al., 2014; Van Ginkel et al., 2018). This linearization can be achieved by translocating the polypeptide chain through a nanopore (Alfaro et al., 2021) or by using a fluorescently labeled motor protein (Yao et al., 2015) to recognize the modified residues required for fingerprinting. Alternatively, the protein fingerprint can be obtained by labeling certain amino acids and determining their location through several Edman degradation cycles (Swaminathan et al., 2018). Although full-length proteins are difficult to analyze owing to the limited number of Edman cycles that can be performed, its utility for analyzing shorter peptides has been shown in a proof of concept. All these approaches have in common that they probe each protein only once, while the accuracy would increase if the same molecule could be measured multiple times.

In this study, we present a protein fingerprinting method that builds further on the concept of residue-specific labeling of selected amino acids and obtains a protein fingerprint by determining the location of amino acids in the 3D structure of a protein. As the size of most proteins lies in the low-nanometer range, our protein fingerprinting approach requires a technique that can determine the location of residues with sub-nanometer resolution. Single-molecule FRET is well suited for this task and comes with the benefit that several thousands of molecules can be imaged at the same time, if full-length proteins can be immobilized in a microfluidic chamber (Lerner et al., 2021). Here we verify the feasibility of a single-molecule FRET-based protein fingerprinting method. We first demonstrate that experimentally obtained fingerprints for four model peptides are distinct and are reproduced by our simulation method. Then we show that simulated fingerprints of 311 human proteome constituents can be identified with 95% accuracy. If mislabeling of residues is assumed to occur, this accuracy decreases to 91%. This supports the notion that FRET X fingerprinting allows for the reliable identification of proteins in complex mixtures.

### Approach

#### FRET X for protein fingerprinting

To realize protein fingerprinting using single-molecule FRET, a resolution sufficient to determine the location of multiple amino acids in the protein structure is required. However, single-molecule FRET analysis is limited to just one or two FRET pairs in a single measurement (Hohng et al., 2004; Clamme and Deniz, 2005). Recently, our group developed a concept to allow for the detection of multiple FRET pairs in a single nanoscopic object. Our technique, FRET X (FRET via DNA eXchange), employs transient hybridization of DNA strands labeled with a fluorophore to temporally separate FRET events that originate from different FRET pairs. We have shown that FRET X can resolve the distance between multiple FRET pairs with sub-nanometer accuracy (Filius et al., 2021; Kim et al., 2021). Here, we apply FRET X for protein fingerprinting. By detecting target amino acids one by one, FRET X produces a unique fingerprint, allowing identification of the protein from a reference database.

**Figure 1:**
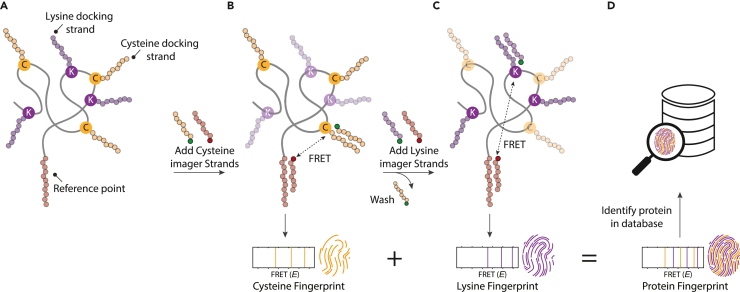
The concept of FRET X for protein fingerprinting (A) A subset of amino acids (here cysteines and lysines) are labeled with orthogonal DNA sequences that function as docking sites for complementary, fluorescently labeled imager strands. Another orthogonal DNA sequence is conjugated to one of the protein termini, which serves as an acceptor docking site and facilitates immobilization of the protein to a microfluidic device. (B) In the first round of FRET X imaging, imager strands that hybridize with the cysteine docking site (yellow circles) and those that hybridize with the reference point (red circles) are injected in the microfluidic chamber. Both the donor and acceptor-labeled imager strands transiently interact with their complementary docking strands. When both are present at the same time, FRET can occur and the FRET efficiency is determined between a cysteine and the reference point. Each of the three FRET pairs is separately probed, giving rise to a number of FRET efficiencies (*E*), which constitute the cysteine fingerprint. (C) The chamber is washed and FRET X imaging is repeated to probe the lysines. This FRET X cycle can be repeated to probe additional amino acids and generate additional fingerprints. (D) The FRET efficiencies for individual amino acids are combined to produce a protein fingerprint that can be mapped against a reference database to identify the protein.

Figure 1 illustrates the workflow for protein fingerprinting using FRET X. A subset of amino acids of a protein of interest is labeled with orthogonal DNA sequences, which serve as docking strands for their complementary imager strands (Figure 1A). One of the protein termini is labeled with a unique DNA sequence, which functions as a reference point and facilitates immobilization of the full-length protein to a microfluidic chip. To obtain a FRET X fingerprint for one of the amino acids, fluorescently labeled imager strands for the terminal reference sequence and for the particular amino acid (e.g., Cysteine, Figure 1B) are added. The imager strands for the reference point are labeled with an acceptor fluorophore, whereas those for the cysteines carry a donor. FRET can occur only when both imager strands are simultaneously bound. The transient and repetitive binding of imager strands reports on the relative location of a residue to the reference point. Furthermore, since the pool of fluorophores is continuously replenished, the effect of photobleaching is mitigated and we can probe each residue multiple times, thereby increasing the precision. After obtaining a sufficient number of FRET events, the FRET X fingerprint can be constructed, reporting on the distance of each target amino acid to the reference point. Then the microfluidic chamber is washed and a new imaging solution is injected to probe a second amino acid (e.g., Lysine) (Figure 1C). The FRET X cycle can be repeated for any number of different amino acids, as long as they are labeled with orthogonal DNA docking sequences. The detection of multiple types of amino acids improves the uniqueness of a protein fingerprint, thereby enhancing the chance of identification. The resolved FRET efficiencies for each amino acid are combined to generate a protein fingerprint, with which a protein can be identified from a reference database (Figure 1D).

#### Fingerprinting simulations

The usefulness of our method hinges on its ability to discern FRET X fingerprints derived from many different proteins, and we run simulations to assess this. Simulating the FRET X fingerprint for a given protein is a complex endeavor, as the fingerprint incorporates both sequence and structural information. Although protein structure prediction has seen major advancements recently, cutting-edge methods (Senior et al., 2020; Xu et al., 2021) remain too computationally costly to assess many proteins. Furthermore, they cannot account for the presence of conjugated DNA tags. Instead, we opted to use a computationally much less intensive lattice modeling approach (Kolinski and Skolnick 2004), in which each residue is represented as a single pseudo-atom, restricted in space to only occupy the vertices of a lattice (Figure S7). Structures are assigned an energy that is lower for structures more likely to occur *in vitro*. Pseudo-atoms may interact with the solvent or with pseudo-atoms on adjacent vertices, incurring either energy bonuses or penalties depending on the residue types involved. A structure can then be efficiently energy minimized using a Markov chain Monte Carlo process. That is, random modifications to the structure are proposed (Figure S9), and for each modification the incurred change in energy determines the probability of accepting it. Despite their simplicity, past investigations have shown that lattice models can reproduce native protein folding behavior (Abeln et al., 2014; Coluzza et al., 2003; Bianco et al., 2017; Dijkstra et al., 2018; Van Gils et al., 2020).

The attachment of DNA tags to selected residues, as required to accurately model our approach, has not previously been included in lattice models. Coarse-grained models have been used to study the effect of dyes linked directly to residues using short linkers, which were found to be minor (Chekmarev, 2019; Allen and Paci, 2010); however, the additional effect of the longer, bulkier DNA tags on structure may be more significant. Although data on DNA-tag-protein interaction is lacking, we find that implementation at the coarse granularity required by lattice models may be built on two basic assumptions: that tags require sufficient unoccupied space to avoid steric hindrance and that they repel each other if situated closely together. Indeed, similar assumptions may be found in other models of ssDNA interaction (Pal and Levy, 2019). A residue marked as tagged loses its ability to interact with other residues and is outfitted with a long, bulky side chain (Figure S11), which incurs heavy energy penalties for clashes with the main structure and attempts to orient itself away from nearby tags.

In the lattice models thus produced, FRET values can then be estimated from the simulated dye positions. To simulate the readout of FRET efficiencies at a given resolution, we bin efficiencies using the resolution as bin width. As we have shown in previous work that a resolution of one FRET percentage point (0.01 E) is achievable, we set the resolution of fingerprints to 0.01 E in simulations, unless otherwise noted. As FRET X allows for orthogonal readout of multiple residue types, the sampling can be repeated to produce the FRET X fingerprints associated with different residue types. Analogously to experimentally obtained fingerprints, simulated FRET X fingerprints for several residue types are then combined to serve as features for automated classification algorithms.

The simulation and classification procedures are described in more detail in the STAR Methods section.

## Results

### Experimental FRET X fingerprinting of model peptides

**Figure 2:**
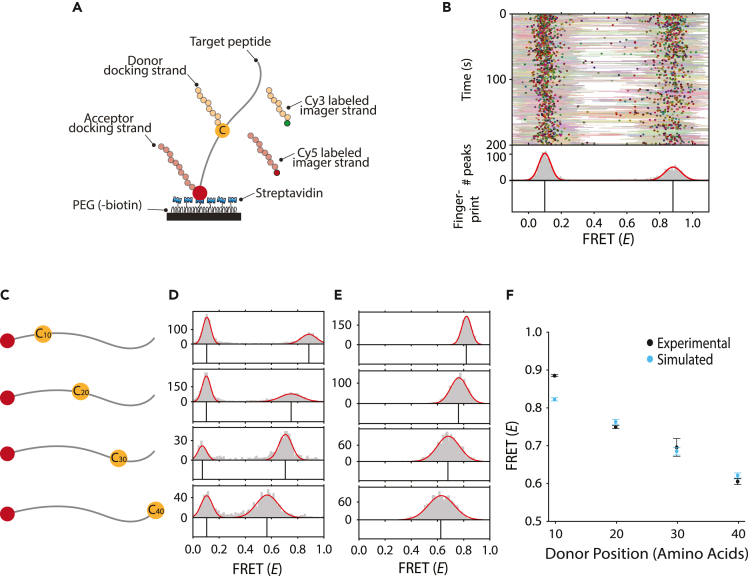
Model peptides can be fingerprinted with FRET X (A) Depiction of the experimental system for peptide fingerprinting. The target peptide is immobilized through conjugation of its N-terminal biotin with the streptavidin on the PEGylated surface. The donor (Cy3)-labeled imager strand (yellow) can bind to the DNA-docking site on the cysteine, while the acceptor (Cy5)-labeled imager strand (red) can hybridize to the docking site on the lysine. Simultaneous binding generates short FRET events and is observed with total internal reflection microscopy. (B) Representative kymograph for a peptide with a cysteine that is 10 amino acids separated from the acceptor-binding site. The FRET efficiency for each data point in a binding event (lines) and the mean FRET efficiency from all data points in a binding event (dots) are indicated as a function of time. A Gaussian distribution (0.88 ± 0.14) is fitted on a histogram of average FRET efficiencies per FRET event. The means of the Gaussians are plotted in a separate panel (bottom) and are referred to as the FRET X fingerprint of the peptide. The FRET population on the left is caused by donor leakage into the acceptor channel. (C) Our four model peptides have a lysine at the N terminus and a cysteine at position 10, 20, 30 or 40. See Table S1 for the full amino acid sequences of the model peptides. (D) Experimental distributions and fingerprints for each peptide show a downward trend in mean FRET (*E*) for increasing FRET pair separation (mean ± FWHM of the Gaussian fit: 0.89 ± 0.14, 0.75 ± 0.20, 0.72 ± 0.11, 0.57 ± 0.20). See also Figures S2 and S3 for imager strand dwell times and kymographs for single peptides, respectively. (E) The simulated distributions and fingerprints for the four peptides show a similar downward trend in distribution means (0.82 ± 0.08, 0.76 ± 0.15, 0.68 ± 0.20, 0.62 ± 0.23). (F) Experimental and simulated data correlate well. Whiskers denote ± one standard deviation. Standard deviation of experimental data points is over four kymographs (each consisting of hundreds of events). Experiments were performed on separate days.

To demonstrate the concept of protein fingerprinting using FRET X and to compare results with computational predictions, we designed an assay where DNA-labeled peptides were immobilized on a PEGylated quartz surface via biotin-streptavidin conjugation (Figure 2A). Each peptide contains an N-terminal lysine for the attachment of a DNA-docking strand, to allow for the transient binding of an acceptor (Cy5)-labeled imager strand. In addition, an orthogonal DNA-docking strand was conjugated to a cysteine residue in the peptide to facilitate transient binding of the donor (Cy3)-labeled imager strands (Figure 2A). The donor and acceptor imager strands were designed to exhibit a dwell time of ∼2 s (Figure S2), so that dyes could be frequently replenished. Furthermore, to increase the probability of the presence of the acceptor imager strand upon donor imager strand binding and allow for FRET detection, we injected 10-fold molar excess of the acceptor imager strand over the donor imager strand. Short-lived FRET events were recorded with single-molecule total internal reflection microscopy upon binding of both donor and acceptor-labeled imager strands to the immobilized target peptide.

Next, we plotted a kymograph to visualize the FRET efficiency of each binding event in a target peptide (Figure 2B). The FRET efficiency for each data point (Figure 2B, lines) and the mean efficiency per binding event are calculated (Figure 2B, circles). A histogram of the mean FRET efficiency per binding event shows distinct FRET populations. Gaussian distributions were fit to resolve peak centers with high resolution (Clamme and Deniz 2005), which together constitute the fingerprint of the peptide (Figure 2B, bottom panel).

To demonstrate the ability of FRET X to distinguish different peptides with varying FRET pair separations, we designed four model peptides. These peptides had an incrementing distance, in steps of 10 amino acids, between donor and acceptor docking strands (Figure 2C). First, we performed single-molecule experiments to obtain experimental FRET X fingerprints and found a clearly discernible peak for each peptide (Figures 2D and S3). Then we simulated FRET X fingerprints for the same sequences using our simulation pipeline and found a similar trend. We only fine-tuned the parameters for the repulsion effect between tags to minimize the difference with experimental values (Figure 2E). Although each histogram showed a wide distribution (full-width half-maximum [FWHM] of ∼0.1–0.2, Figures 2D and 2E), the Gaussian fit can be used to resolve the peak with high precision of <0.01 (standard error of mean), where the achievable precision depends on the number of binding events (Filius et al., 2021). Furthermore, in both simulations and experiments we observed a monotonous decrease in FRET efficiency for increasing FRET pair separation. Furthermore, the experimentally obtained fingerprints generally correlate well with values found by simulations (Figure 2F). Since for each peptide the minimum inter-peptide difference in FRET (*E*) is larger than the maximum standard deviation, we find that we can distinguish these four peptides by their FRET X fingerprint.

### Fingerprinting simulation of protein spliceoforms

**Figure 3:**
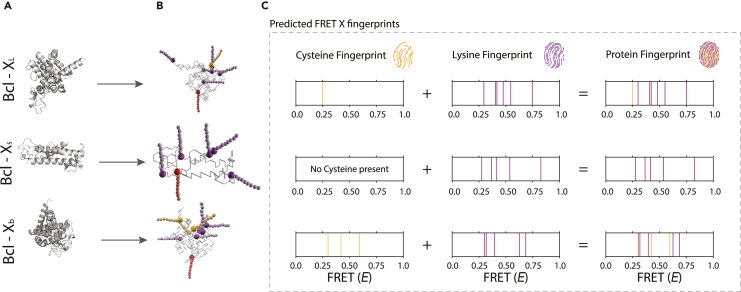
Representative FRET (*E*) fingerprints for three spliceoforms of BCL-X (A) Fully atomic structure for BCL XL, Xs, and Xb (from top to bottom) as predicted by the RaptorX structure prediction tool (Källberg et al., 2012, 2014). (B) Energy-optimized lattice model structures with DNA-docking strands attached to cysteines (orange) and lysines (purple). The reference acceptor docking strand (red) is added to the N terminus of the proteins. (C) The simulated fingerprint for spliceoform of the BCL proteins. Fingerprints are based on averaged donor-acceptor distances in 100 structural snapshots of Markov chain-generated lattice model structures (distributions shown in Figure S4). Fingerprints for a second set of spliceoforms (PTGS1) are shown in Figure S5.

We set out to evaluate the performance of our method for targeted proteomics, based on simulations. For this we sought to identify the different spliceoforms of the apoptosis regulator Bcl-2 (UniProt ID: Q07817), which are potential biomarkers for cancer (Kale et al., 2017) and are likely to produce different fingerprints. BCL-X_L_ is an anti-apoptotic regulator, whereas both Bcl-X_S_ and Bcl-X_b_ are pro-apoptotic factors (Kale et al., 2017; Shiraiwa et al., 1996). The ratio between these factors is important for cell fate. We simulated simultaneous labeling of cysteine (C) and lysine (K) to create C + K fingerprints for each of the spliceoforms, Bcl-X_L_, Bcl-X_S_, and Bcl-X_b_ (Figures 3A and 3B). As the spliceoforms differ in the numbers and locations of C and K residues, we expected their fingerprints to be dissimilar. This was indeed the case in simulation (Figure 3C). Fingerprints do vary across individual molecules of the same spliceoform; however, the fingerprints remain sufficiently characteristic to identify each spliceoform by eye (Figure S5A). We also trained and tested a support vector machine (SVM) classifier on 10 replicates in a 10-fold cross-validation scheme and attained an accuracy of 100%.

We then simulated a more difficult scenario, in which we attempted to classify fingerprints for six spliceoforms of PTGS1 (UniProt ID: P23219) (Garcia-blanco et al., 2004). Although the higher number of C and K residues made discrimination of fingerprints by eye harder, an SVM trained and tested in a 10-fold cross-validation scheme was still able to separate the six spliceoforms with 100% accuracy (Figure S5B).

### Analysis of simulated protein mixtures

To evaluate a test case displaying a complexity closer to that found in a single cell, we selected all UniProt human proteome (ID: UP000005640) entries that were linked to a single-chain structure in the RCSB protein database and for which lattice modeling was able to find a configuration without steric hindrance of docking strands (n = 311). Based on available targeted residue labeling chemistries and relative residue frequencies in naturally occurring proteins, we simulated labeling schemes involving cysteine (C), lysine (K), and arginine (R). For each protein we generated fingerprints based on 10 separately simulated molecules, after which we trained and tested an SVM classifier in a 10-fold cross-validation scheme. Here we measure overall classifier accuracy. To identify the subset of proteins for which our method works well, we also analyzed the number of well-identifiable proteins, i.e., those for which more than five of the replicates were identified correctly.

**Figure 4:**
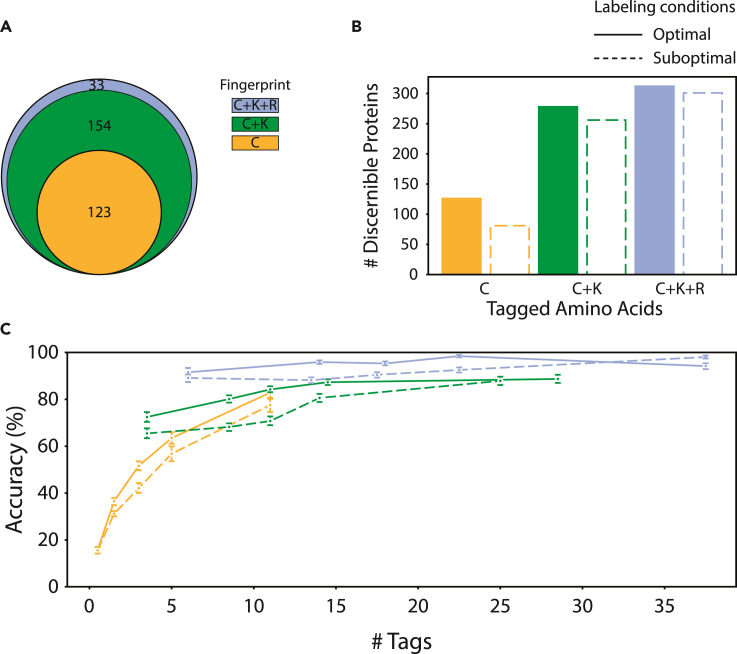
FRET X fingerprinting simulation results assuming optimal and suboptimal experimental conditions FRET X fingerprint classifier cross-validation performance measures are shown for three combinations of tagged residue types, C, C + K, and C + K + R, and two labeling qualities, “optimal,” where all targeted residues and no off-target residues were labeled, and “suboptimal,” where erroneous labeling occurred following the rules in Table S3. (A) Venn diagram showing numbers of proteins that were found to be well identifiable, i.e., that were correctly identified in more than 5 of 10 cross-validation folds. The total number of proteins is 311. (B) The identification accuracy of proteins under optimal and suboptimal labeling conditions. (C) Average classifier accuracy as a function of the number of tagged residues in structures, aggregated in five groups with similar numbers of tags. Whiskers denote ± one standard deviation. Accuracies for different resolutions and fsuboptimal labeling scenarios are shown in Figure S6.

We find that our classifier performs at 45% accuracy on C-labeled proteins. Of 311 proteins, 126 were well identifiable, indicating that labeling only C residues is sufficient to consistently recognize this subset of proteins (Figure 4A, orange circle). Fifty-seven proteins did not contain C residues and are thus impossible to identify using only C labeling. The remaining 130 poorly identifiable proteins generally produced fingerprints containing few FRET values or highly variable fingerprints, the latter indicating a lack of structure stability.

When C + K or C + K + R residues were labeled, accuracy rose to 82% and 95%, respectively (Figure 4B). As expected, fingerprints are more likely to obtain a characteristic signature if distances for more residue types are tracked. Numbers of well-identifiable fingerprints also rose to 278 and 312 of 311, respectively. Regardless of which residue types are labeled, we find that proteins containing more tagged residues can be identified with higher accuracy (Figure 4C).

### Robustness against suboptimal experimental conditions

To investigate the effect of labeling errors, we ran simulations for a suboptimal labeling scenario, with a 90% probability of labeling the target residue and a certain non-zero probability to label non-target residues (C: 1%, K:1%, R:0.5%, Table S3). For C and K these probabilities were based on experimentally determined efficiencies and specificities found in the literature (Boutureira and Bernardes, 2015; Abello et al., 2007; Thompson et al., 2016).

Overall, we find that labeling errors incur a modest decrease in classifier performance; for C, C + K, and C + K + R labeling, accuracy drops from 45%, 82%, and 95% to 39%, 74%, and 91%, respectively (Figure 4B). This indicates that FRET X fingerprints, particularly those gained from C + K + R labeling, contain the redundant information required to mitigate the effect of imperfect labeling (Figure 4C). We also investigated the effect of decreased measurement resolution; however, only after reducing resolution far beyond experimentally attainable levels, past 0.10 E, did we find severe reductions in accuracy (Figure S6).

## Discussion

Here we present a protein fingerprinting approach that determines the location of amino acids within a protein structure using FRET X. We provide evidence of its ability to identify proteins in heterogeneous mixtures using simulations and demonstrate its technical feasibility by producing experimental fingerprints for designed peptides.

We experimentally demonstrate fingerprinting of peptides of 40 amino acids and observe a monotonous decrease in FRET efficiency. This trend is supported by simulations and suggests that our model peptide has a relatively linear conformation. These peptides do not exhaust the lower end of the FRET-efficiency domain, which implies that larger peptides and proteins with increased FRET pair separation can be fingerprinted. Although most proteins are considerably larger than 40 amino acids, they usually adopt a globular structure, which reduces the FRET pair separation. The average protein is estimated to have a diameter of 5 nm (Erickson, 2009), whereas the FRET dyes (Cy3-Cy5) used here are expected to be accurate at distances of up to ∼7 nm (Lerner et al., 2021). Therefore, our FRET X fingerprinting approach could be suitable for the identification of a large set of human proteins. This notion is substantiated by the simulations run using our lattice model, which shows that also for larger proteins the FRET X fingerprints remain discernible.

We show that simulated fingerprints are sufficiently unique and reproducible to consistently identify the majority of the proteins in our simulation pool. Moreover, this result could be achieved by labeling up to three types of amino acids: cysteine, lysine, and arginine, all of which can be targeted for specific labeling using existing chemistries (Alfaro et al., 2021; Boutureira and Bernardes, 2015; Abello et al., 2007; Thompson et al., 2016). Of interest, even if only cysteine is labeled we find that a considerable subset of proteins remained consistently identifiable, although labeling additional residue types does increase accuracy, the number of identifiable proteins, and robustness against labeling errors. It should also be noted that the set of residue types targeted for FRET X fingerprinting can be expanded even further; labeling of, e.g., methionine (Lin et al., 2017) or tyrosine (Alvarez Dorta et al., 2020) may be employed to further increase accuracy or tailor our method to the detection of a given target protein.

A far-reaching goal of the proteomic community is to detect and analyze all proteoforms that can be derived from a single protein encoding gene (Aebersold et al., 2018). Most proteoforms have subtle differences, e.g., alternative splicing or post-translational modification, and are difficult to detect with current technologies, such as ELISA, MS, or native MS (Leney and Heck, 2017). We have shown that FRET X has the ability to distinguish peptides based on the location of a single cysteine, a subtlety akin to those found in many isoforms, and we have shown two cases in which clinically relevant spliceoforms are well distinguishable based on their simulated FRET X fingerprints. This suggests that our FRET X fingerprinting platform would be a suitable complementary technique for the detection of clinically relevant proteoforms.

### Limitations of study

Although care has been taken to account for the effects of our experimental method on target protein structures, and thus the produced fingerprints, we note that the nature of several potentially influential factors has yet to be elucidated. For our simulations we investigated proteins for which the structure had already been determined; however, in our experimental system, a microfluidic chamber with non-physiological conditions, proteins may adopt a different structure or a set of several different structures, creating a discrepancy between simulated and experimental fingerprints. Furthermore, although we model the effects of lower labeling efficiency and specificity, we have insufficient information to model how adjacency of residues targeted for labeling will affect efficiency of labeling chemistries. Once proteins can be fingerprinted more routinely, more data will be available to support modeling choices accounting for these factors. We stress that it is primarily the uniqueness and reproducibility of a fingerprint that is important for protein identification, not necessarily its predictability from a known structure. Although our current simulations were performed on a set of 311 known protein structures, we envision that the number of proteins that can be fingerprinted using our FRET X approach will increase significantly owing to recent developments in protein structure prediction tools (Xu et al., 2021; Jumper et al., 2021; Tunyasuvunakool et al., 2021). Furthermore, we expect that, as the diversity of a sample decreases from several hundreds to tens of different proteins through sample fractionation, the fingerprint uniqueness and thereby the fraction of correctly identified proteins sharply increases. Adequate sample preparation and purification to reduce sample complexity will be important for more targeted approaches.

## STAR★Methods

### Key resources table

**Table:** 

REAGENT or RESOURCE	SOURCE	IDENTIFIER
**Chemicals, peptides, and recombinant proteins**		

mPEG (MW 5,000)	Lysan	Cat# MPEG-SVA-5000-1g
mPEG-biotin (MW 5,000)	Lysan	Cat# Biotin-PEG-SVA-100mg
Amino-silane	Sigma	Cat# 281778
MS4-PEG	Thermo Fisher Scientific	Cat# 22341
Streptavidin	Invitrogen	Cat# S-888
Glucose oxidase	Sigma	Cat# G2133
TROLOX	Sigma	Cat# 238813
Catalase	Roche	Cat# 10106810001
Tris(2-carboethyl)phosphine	Sigma	Cat#646547
Dimethyl sulfoxide	Sigma	Cat#276855
Dibenzocyclooctyne-*N*-hydroxysuccinimidyl ester	Sigma	Cat#761524
Pierce™ C18 Tips, 10 μL bed	ThermoFisher Scientific	Cat#87782
Model Peptides, See Table S1	This Study	N/A

**Deposited data**		

Lattice models and simulated fingerprints	This study	https://doi.org/10.5281/zenodo.5330741

**Oligonucleotides**		

See Table S2	This Study	N/A

**Software and algorithms**		

IDL (ITT visual information solutions)	http://www.harrisgeospatial.com/	N/A
Simulation code	This study	https://doi.org/10.5281/zenodo.5330741
Data analysis code	https://github.com/kahutia/transient_FRET_analyzer2	N/A

### Resource availability

#### Lead contact

Further information and requests for resources and reagents should be directed to and will be fulfilled by the lead contact, Dick de Ridder (dick.deridder@wur.nl).

#### Materials availability

• 40-residue model peptide sequences are given in Table S1.
• Imager and docking strand sequences are given in Table S2.

### Method details

#### Peptide labeling

Custom designed polypeptides were obtained from Biomatik (Canada) and had a constant backbone sequence (Table S1), differing only in the cysteine substitutions. Cysteine residues of the polypeptides were reduced with 40-fold molar excess Tris(2-carboethyl)phosphine (TCEP) for 30 min and then donor-labeled with 6-fold molar excess monoreactive maleimide-(5′) functionalized DNA in 50 mM HEPES pH 6.9 overnight at room temperature. The acceptor docking strand was labeled onto a single lysine that is located at the N-terminus of the peptide. For this, Dimethyl sulfoxide (DMSO) was added to 50% (v/v) and the pH was increased to pH 7.5 through the addition of NaOH. Next, we added monoreactive N-Hydroxysuccinimide (NHS)-ester functionalized Dibenzocyclooctyne (DBCO) (Sigma Aldrich, Germany) in a 25-fold molar excess and incubated for 6 hours at room temperature. Free NHS-DBCO was removed by using C18 bed micropipet tips (Pierce) according to manufacturer's protocol. Finally, monoreactive Azidobenzoate-(5′) functionalized-DNA was added in 5-fold molar excess and incubated overnight at room temperature. See Tables S1 and S2 for the full list of substrates.

#### Single-molecule setup

All experiments were performed on a custom-built microscope setup. An inverted microscope (IX73, Olympus) with prism-based total internal reflection was used. In combination with a 532 nm diode-pumped solid-state laser (Compass 215M/50mW, Coherent). A 60× water immersion objective (UPLSAPO60XW, Olympus) was used for the collection of photons from the Cy3 and Cy5 dyes on the surface, after which a 532 nm long pass filter (LDP01-532RU-25, Semrock) blocks the excitation light. A dichroic mirror (635 dcxr, Chroma) separates the fluorescence signal which is then projected onto an EM-CCD camera (iXon Ultra, DU-897U-CS0-#BV, Andor Technology). A series of EM-CDD images was recorded using a custom-made program in Visual C++ (Microsoft).

#### Single-molecule data acquisition

Single-molecule flow cells were prepared as previously described (Chandradoss et al., 2014; Filius et al., 2020). In brief, to avoid non-specific binding, quartz slides (G. Finkerbeiner Inc) were acidic piranha etched and passivated twice with polyethylene glycol (PEG). The first round of PEGylation was performed with mPEG-SVA (Laysan Bio) and PEG-biotin (Laysan Bio), followed by a second round of PEGylation with MS(PEG)_4_ (ThermoFisher). After assembly of a microfluidic chamber, the slides were incubated with 20 μL of 0.1 mg/mL streptavidin (Thermofisher) for 2 minutes. Excess streptavidin was removed with 100 μL T50 (50mM Tris-HCl, pH 8.0, 50 mM NaCl). Next, 50 μL of 75 pM DNA-labeled peptide was added to the microfluidic chamber. After 2 minutes of incubation, unbound peptide and excess Azide-DNA from the earlier click reaction was washed away with 200 μL T50. Then, 50 μL of 10 nM donor labeled imager strands and 100 nM acceptor labeled imager strands in imaging buffer (50 mM Tris-HCl, pH 8.0, 500 mM NaCl, 0.8% glucose, 0.5 mg/mL glucose oxidase (Sigma), 85 ug/mL catalase (Merck) and 1 mM Trolox (Sigma)) was injected. All single-molecule FRET experiments were performed at room temperature (23 ± 2°C).

#### Data analysis

Fluorescence signals are collected at 0.1-s exposure time unless otherwise specified. Time traces were subsequently extracted through IDL software using a custom script. Through a mapping file, the script collects the individual intensity hotspots in the acceptor channel and pairs them with intensity hotspots in the donor channel, after which the time traces are extracted. During the acquisition of the movie, the green laser is used to excite the Cy3 donor fluorophores. For automated detection of individual fluorescence imager strand binding events, we used a custom Python code (Python 3.7, Python Software Foundation, https://www.python.org) utilizing a two-state K-means clustering algorithm on the sum of the donor and acceptor fluorescence intensities of individual molecules to identify the frames with high intensities (Boutureira and Bernardes, 2015). To avoid false positive detections, only binding events that lasted for more than three consecutive frames were selected for further analysis. FRET efficiencies for each imager strand binding event were calculated and used to build the FRET kymograph and histogram. Populations in the FRET histogram are automatically classified by Gaussian mixture modeling. The automated analysis code in Python is freely available at: https://github.com/kahutia/transient_FRET_analyzer2.

#### Simulations

Fingerprinting simulations were generated using a lattice folding model written in Python 3.7, on. Simulation and analysis code are freely available at https://github.com/cvdelannoy/FRET_X_fingerprinting_simulation. To run simulations, python and conda installations are required.

A protein folding simulation was implemented to incorporate DNA-tags attached to certain residues and account for their effect on the protein structure. Lattice models were used because of the far lower computational power needed for folding simulations compared to fully atomistic models allowing unrestricted movement, which is attained by reducing each amino acid to a pseudo-atom and restricting its possible positions to the vertices of a lattice. Such models have previously been used in applications where low computational requirements were essential (Kolinski and Skolnick, 2004; Abeln et al., 2014; Coluzza et al., 2003; Bianco et al., 2017; Dijkstra et al., 2018; Van Gils et al., 2020). A schematic overview of the simulation pipeline is given in Figure S7. The procedure starts with a fully atomistic native structure, which is converted to a lattice structure with tagged residues marked. This structure is then refolded by making local modifications and calculating the effect these have on the model energy (*E*_*tot*_), as calculated by an energy function. Modifications that decrease *E*_*tot*_ are accepted, whereas those that increase *E*_*tot*_ are more likely to be discarded the more they increase *E*_*tot*_. The procedure ends when all DNA-tags fit in the structure without causing steric hindrance. Aspects of the modeling procedure are described in more detail below.

#### Lattice structure

The lattice modeling procedure employed here largely resembles those in previously published applications. In particular, the model developed by Abeln et al. (2014) was used as a starting point, however the cubic lattice was replaced by a body-centered cubic (BCC) lattice (Figure S1). The octahedral unit cell of a BCC lattice borders eight neighboring cells through its hexagonal faces and four through its square faces. Only connections through hexagonal faces are considered, as this allows all bonds to be of the same length. As a result, only even coordinates in the lattice are valid vertices for residue placement (Thompson et al., 2016). This implementation increases the number of contacts that each non-endpoint residue can make from four to six (not including immediately neighboring residues) and increases the number of directions into which a bond may extend. The resulting increased flexibility allows lattice models to more closely resemble native folds. Moreover, alpha helices are represented better as the BCC lattice allows structures that make one regular turn per five residues.

#### Tag implementation

As the precise effect of the presence of DNA-tags on protein structure is unclear, we relied on several basic assumptions to include them in the model. First, we assume that DNA-tags prefer to reside in the periphery of a protein due to their polar backbones. Thus, labeling an internal residue should alter local structure to accommodate sufficient space from the residue to the surface, while tagging a residue that already resides on the protein surface should affect the structure less severely. This was implemented by adding a substantial energy penalty if a tagged residue did not have space for a DNA tag to reach the periphery of the structure without clashing with the main chain. As DNA nucleotides are bulkier than amino acids, we account for this by modeling the tag with a volume spanning vertices up to 2 vertices away from the tag backbone (Figure S11). Secondly, we assume that tags will electrostatically repel each other. This is represented by introducing a minimum angle and dihedral between tag pairs that are spatially close together in a given configuration (Figure S10). To parameterize this effect, we compared predicted fingerprints of 40-residue model peptides to the presented experimental data and found that values are reproduced well if at least a 70° angle and dihedral are enforced between tags situated within 20 Å of each other. Lastly, as DNA labels obstruct or partially replace the residue, the labeled residue is assumed to lose its ability to interact with other residues or contribute to secondary structure formation, including disulfide bridges in the case of cysteine labeling (Figure S8).

#### Simulated labeling scenarios

Two labeling scenarios are employed in this work. Under the optimal scenario, all target residues are labeled and no off-target labeling takes place. Under the suboptimal scenario, both labeling efficiency and specificity are decreased, following a similar procedure to Ohayon et al. (2019); each target residue has a 90% chance of being labeled by its dedicated chemistry, while some off-target labeling probability is defined for one or more other residue types. Where possible, efficiency and specificity parameters are based on literature (Table S3).

#### Structure collection

We base the lattice models used in our fingerprinting simulations on fully atomistic structures as stored in the RCSB PDB. To obtain a dataset of relevant structures, we analysed all available PDB entries corresponding to entries in the Uniprot human proteome set (UP000005640). Of the 20,381 entries in the proteome, 7,133 solved structures were found. We further filtered this list on structure quality, retaining only those with an R-free value below 0.21, and removed structures with non-canonical residues as our model contains no energy modifiers for these residues. Lastly, quaternary structure is expected to be lost during sample preparation, thus to avoid having to model the effect of losing other chains on the tertiary structure of the target chain, we removed structures which were crystalized as a complex of multiple chains. After these filtering steps, 746 structures remained for our simulations.

A lattice model is derived from a fully atomistic structure by reducing it to its Cα positions and placing each Cα-atom on the nearest lattice vertex, while remaining connected to its neighboring Cα-atom, starting from the residue with the lowest index. Alpha helices are forced to remain intact on the lattice, by first translating involved Cα-atoms to a lattice-compliant helix and then minimizing the distance between their respective lattice positions simultaneously.

As no PDB structures are available for the 40-residue model peptides labeled in practical experiments, starting structures for these peptides were stretched configurations. Starting structures for Bcl-X and PTGS1 spliceoforms were generated using the RaptorX structure prediction server (Källberg et al., 2012, 2014).

#### Folding simulation

After initialization of the lattice model, a Markov Chain Monte Carlo (MCMC) procedure is employed to minimize the structure energy *E*_*tot*_.

$$E_{tot}=E_{AA}+E_{sol}+E_{ss}+E_{dsb}+E_{tag}+E_{reg}$$

Residue interaction and residue-solvent interaction terms *E*_*AA*_ and *E*_*sol*_ are summed pairwise interaction terms between contacting residues or residue-solvent contacts, the magnitudes of which are obtained empirically (Miyazawa and Jernigan, 1999). The secondary structure formation energy term *E*_*ss*_ is adapted from Abeln et al. (2014) and incurs an arbitrarily high energy bonus of −25 if an alpha helix or beta sheet is formed, but only if a given residue also was part of such a secondary structure in the native fold. An alpha helical residue incurs this bonus if the exact shape of the helix is formed (i.e. residue *i* up to *i*+4 take the same relative orientation at each step), while a bonus for beta sheet formation is applied if non-neighboring beta-sheet residues are adjacent to each other. The disulfide bridge energy term *E*_*dsb*_ incurs an arbitrarily high bonus of 50 for each pair of contacting cysteines. Each cysteine may only contribute to one bond at a time. The tag energy term *E*_*tag*_ incurs an arbitrarily high energy penalty of 100 for each residue impeding the shortest route from a tagged residue to the periphery of the structure. Lastly, the regularization term *E*_*reg*_ incurs a penalty for large structural reorganizations occurring in a single MCMC step, as we found that this helps to retain the native fold as much as possible.

To minimize the energy of a structure, three modifications may be applied (Figure S9). A branch rotation modification rotates all pseudo-atoms after a randomly chosen point; a corner flip modification changes the position of a single pseudo-atom to a new position without losing contact with its neighbors; and a crankshaft move does the same for two pseudo-atoms. Note that these modifications are similar to those used by Coluzza et al. (2003), with the difference that we do not restrict angles for corner flips and branch rotations are less constricted, whereas Coluzza et al. (2003) only allow 180- and 90-degree rotations respectively.

At each MCMC iteration of the energy minimization process, one of the mentioned modifications is applied at a random position. The energy difference incurred by the modification determines the probability with which it is accepted; modifications decreasing the model energy or keeping it the same are always accepted, while those increasing the energy may still be accepted with some probability to allow for better exploration of the search space. The probability of modification acceptance is defined as:

$$P_{accept}=min\left\{1,exp\left(\frac{-\Delta E}{T}\right)\right\}$$

Here Δ*E* is the energy difference and *T* is the simulation temperature, a unitless parameter which regulates the probability of acceptance of an energy-increasing modification. Two models are optimized simultaneously in two chains, at two different simulation temperatures (0.01 and 0.001). The high-temperature chain is less restrictive in its acceptance of energy-increasing modifications, thus allowing a wider exploration of the structural search space, while the more stringent acceptance criterion of the low-temperature chain is better at finding a local minimum. The two properties are combined by allowing the chains to exchange models after each 100 MCMC iterations. The probability of exchange is given by:

$$P_{exchange}=min\left\{1,exp\left(\Delta E\cdot \Delta 1/T\right)\right\}$$

Lattice models are optimized for a maximum of 60,000 MCMC iterations using two separate chains. After each 500 iterations, the steric hindrance incurred by DNA-tags is assessed. If no steric hindrance remains, the model is not optimized any further.

#### Fingerprint extraction

To account for the fact that a structure may adopt several conformations over the course of measurements, either due to native disorder or due to the presence of the DNA-tags, fingerprints are based on a series of structure snapshots. After the folding simulation has finished and the structure which accommodates all DNA-tags without steric hindrance is found, another 1,000 MCMC steps are performed. During these steps, snapshots are taken at intervals of 10 steps, thus measuring 100 slightly different conformations. For each snapshot, dye positions are chosen randomly from all accessible lattice directions. If tags are found to be closer than 20Å to each other, a minimum angle and dihedral angle of 70° each between those tags is enforced (Figure S10). Distances between donor and acceptor dye positions are estimated from the snapshots and averaged over 10 consecutive conformations, to emulate the movement of the molecule over a single frame. Using this distance, the FRET efficiency is then calculated as follows:

$$E_{FRET}=\frac{1}{1+{\left(R/R_{0}\right)}^{6}}$$

Here *R* is the modeled distance between donor and acceptor dye and *R*_0_ is the Förster radius, which characterizes the used FRET dye pair (*R*_0_ assumed constant at 54Å for the Cy3-Cy5 FRET pair (Lerner et al., 2021)). Finally, all FRET values are binned and normalized over the number of simulated frames to produce the final fingerprint. The bin width is used here to represent the observation resolution. Resolution is fixed at 0.01 unless otherwise noted, as previous work has shown that such a resolution can be achieved using FRET X (Filius et al., 2021). If multiple residue types are tagged, each residue type generates its own fingerprint which is binned separately.

#### Classification

To classify simulated fingerprints a support vector machine (SVM) was implemented using the scikit-learn package (v0.23.2) (Pedregosa et al., 2011). As a higher resolution is also more sensitive to noise by unstable fingerprints, the resolution is tuned during training in steps of 0.01 *E* to produce the highest training accuracy.

### Quantification and statistical analysis

Statistical analysis details for experimental data are given in the captions of Figures 2D and 2F. Statistical details for simulations of complex samples are found in the results section and Figure 4. We evaluated the SVM classifier’s ability to classify simulated fingerprints in a ten-fold cross validation procedure; the SVM was fitted to a training set consisting of 90% of produced fingerprints and tested on the held-out test set. To evaluate classifier performance, we calculated test accuracy, i.e. the number of correct classifications over total number of test examples. The reported accuracy is the arithmetic mean of the accuracies produced for each fold. As this measure obscures whether classification mistakes are consistently made for certain proteins or are randomly distributed, we also determined which proteins were correctly classified in more than half of replicates, which we denote as well-identifiable proteins.

## Data Availability

Section 1: data• Simulated data have been deposited on Github and are publicly available as of the date of publication. The DOI is listed in the Key resources table. • Experimental data reported in this paper will be shared by the lead contact upon request. Section 2: code• All original code has been deposited on Github and is publicly available as of the date of publication. DOIs are listed in the Key resources table. Section 3: Any additional information required to reanalyze the data reported in this paper is available from the lead contact upon request.
