# Supplementary material for: Evaluation of FRET X for single-molecule protein fingerprinting
Source: iScience. 2021 Oct 5;24(11):103239. doi: 10.1016/j.isci.2021.103239 (PMC8546410; doi:10.1016/j.isci.2021.103239)
Supplement: Document S1. Figures S1–S11 and Tables S1–S3 [file mmc1.pdf]

iScience, Volume *24*

Supplemental information

Evaluation of FRET X for single-molecule protein fingerprinting

Carlos Victor de Lannoy, Mike Filius, Raman van Wee, Chirlmin Joo, and Dick de Ridder

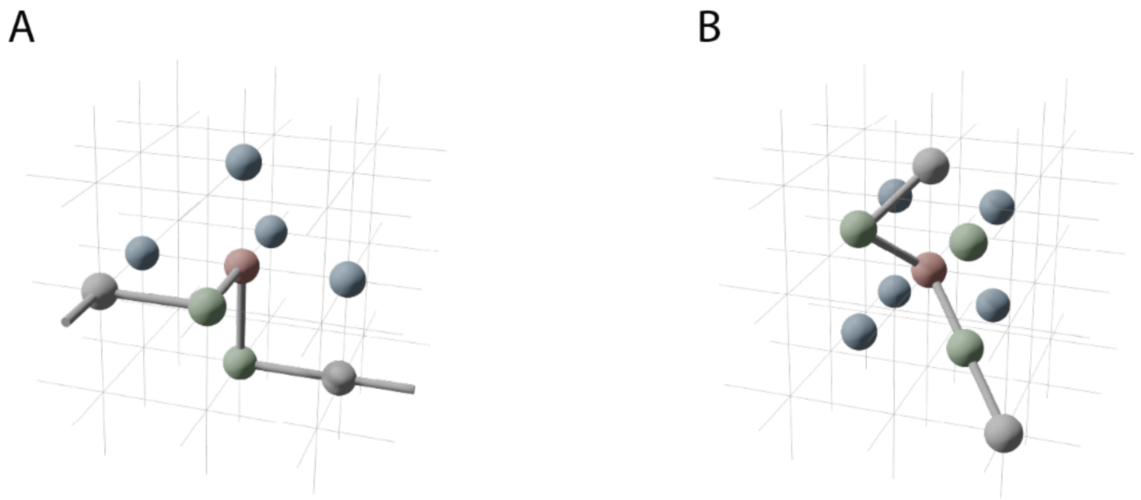

Supplementary figure 1: Pseudo-atoms on a cubic lattice (A) and a body-centered cubic lattice (B). Related to STAR methods.

Shown are one main pseudo-atom (red) and its direct neighbors (green). For the main pseudo-atom, all possible adjacent pseudo-atoms (green) are depicted. Figures were generated in Blender 2.93.0.

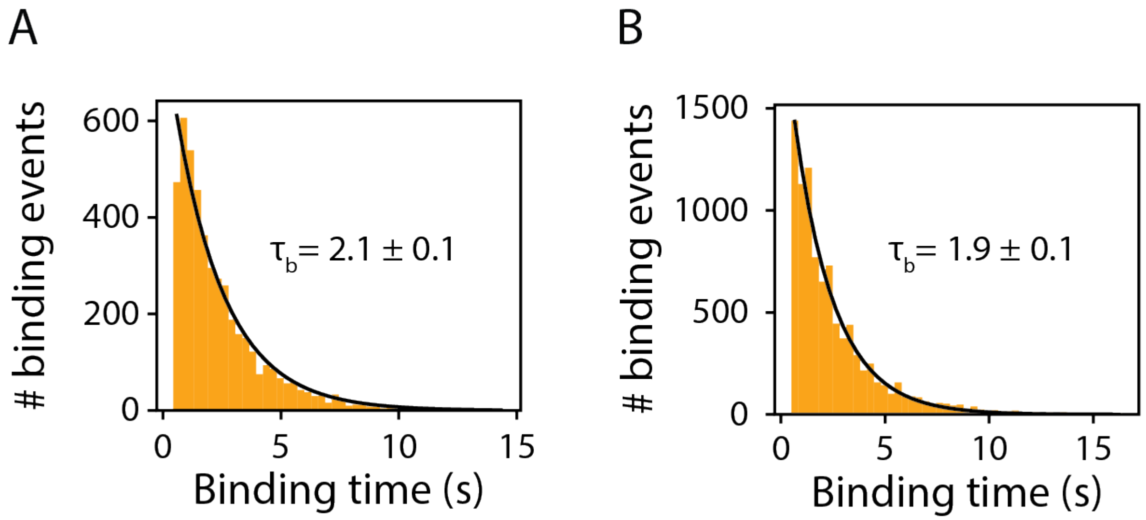

Supplementary figure 2: Single-molecule binding kinetics of FRET X imager strands. Related to figure 2D.

(A) Dwell-time histogram for the FRET X donor imager strand **(Supplementary Table 2)** fitted with a maximum likelihood estimation for a single exponential distribution (black line). Average ± standard deviation of four different estimations gives: 2.1 ± 0.1 s. The number of datapoints for this distribution: n = 4687 and peptide used was K1C40. **(B)** Dwell-time histogram for the FRET X acceptor imager strand **(Supplementary Table 2)** fitted with a maximum likelihood estimation for a single exponential distribution (black line). Average ± standard deviation of four different estimations gives: 1.9 ± 0.1 s. The number of datapoints for this distribution: n = 9477 and peptide used was K1C40.

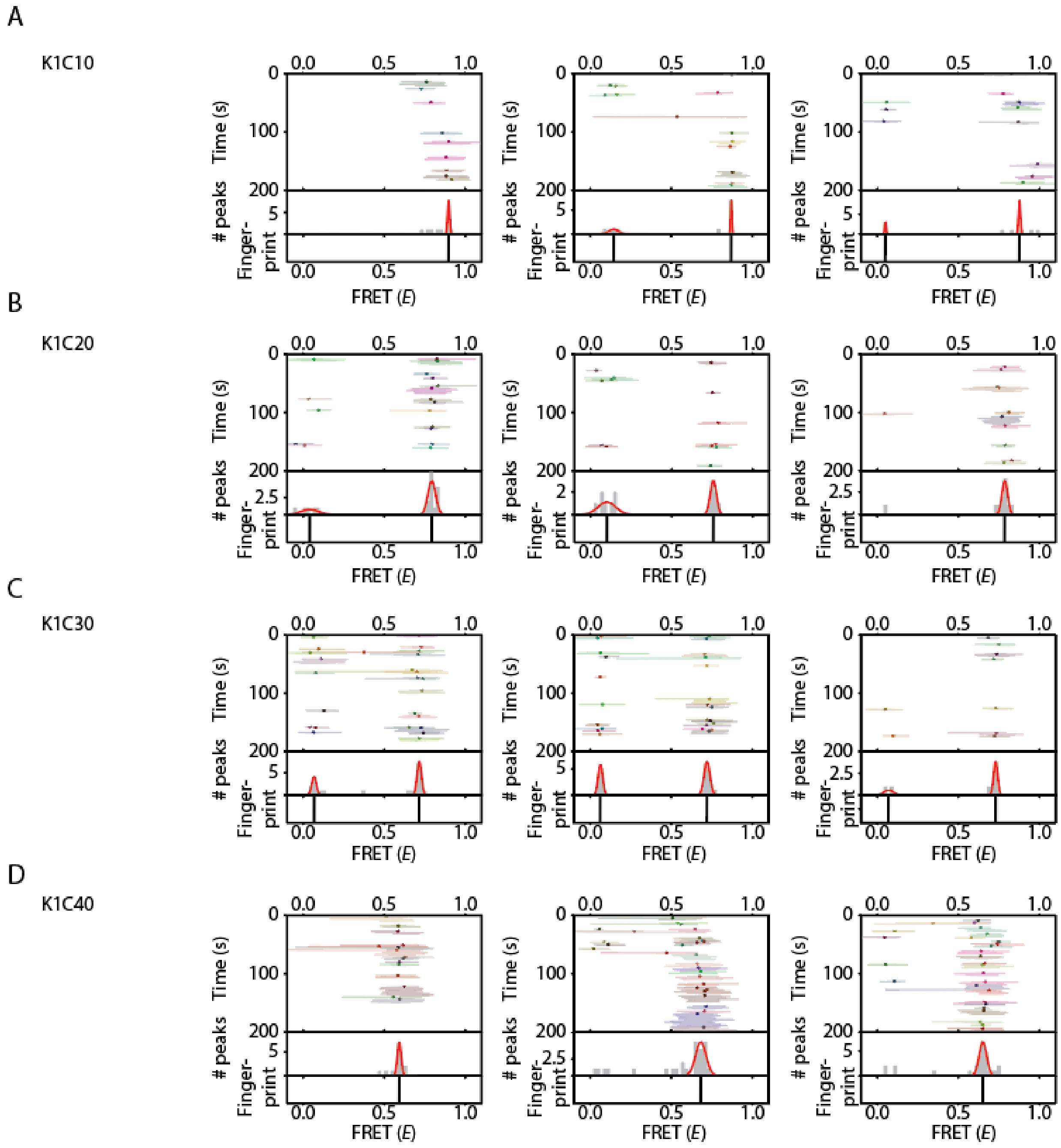

Supplementary figure 3: Representative kymographs of individual peptides. Related to figure 2D.

(A-D) Representative single-molecule FRET kymographs for each of the four peptides. The downward FRET (*E*) trend remains at the single-molecule level and the distribution of each individual molecule can be fitted with high precision (s.d. ≤ 0.03 for each distribution). The ensemble of many identical single-molecules results in the FRET-fingerprint (**Figure 2B**).

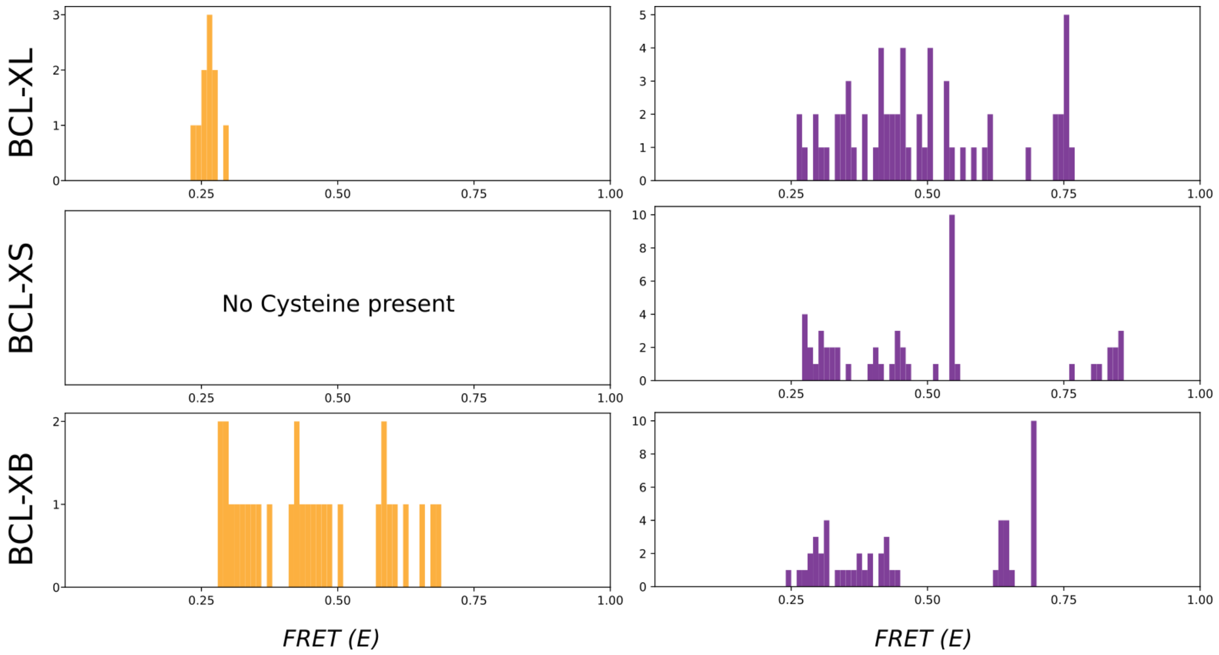

Supplementary figure 4: Histograms of simulated FRET events for a single molecule each of three Bcl spliceoforms. Related to figure 3. Cysteine and lysine-derived values are colored orange and purple respectively**.** The corresponding FRET X fingerprints, for which FRET values are averaged per tagged residue, are shown in Figure 3C.

A

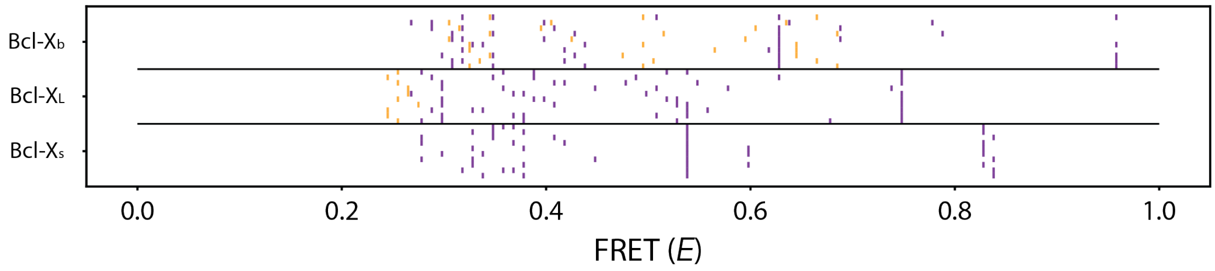

B

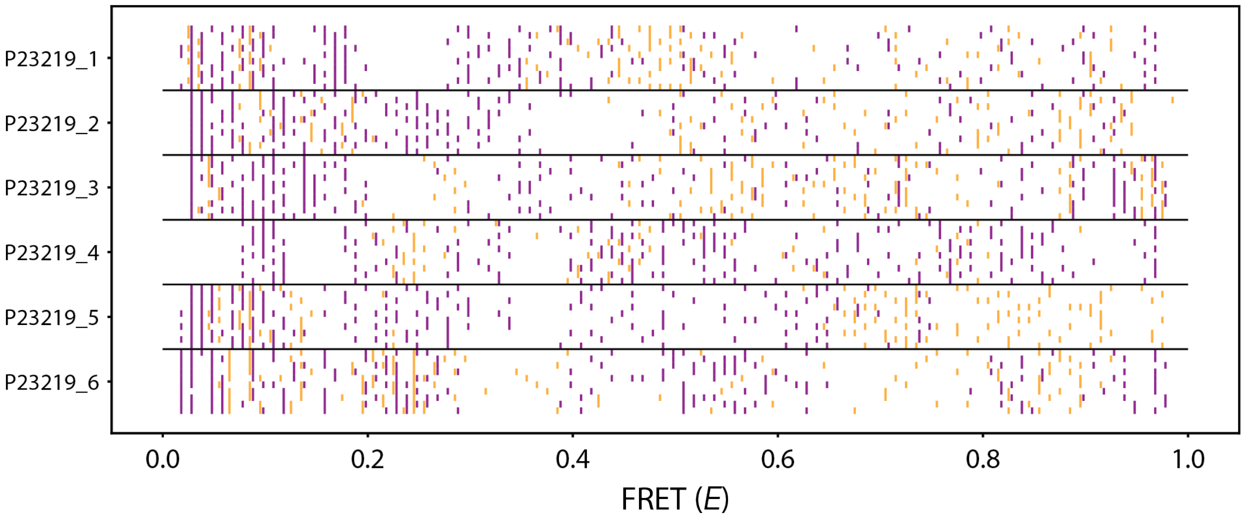

Supplementary figure 5: Simulated FRET X fingerprints for spliceoforms of Bcl and PTGS1. Related to figure 3. (A) FRET X fingerprints for ten simulated molecules, one per horizontal line, of three Bcl-X spliceoforms: Bcl-X_L, Bcl-X_S and Bcl-X_B. Cysteine and lysine-derived values are colored orange and purple respectively**. (B)** FRET X fingerprints for ten simulated molecules of six PTGS1 spliceoforms.

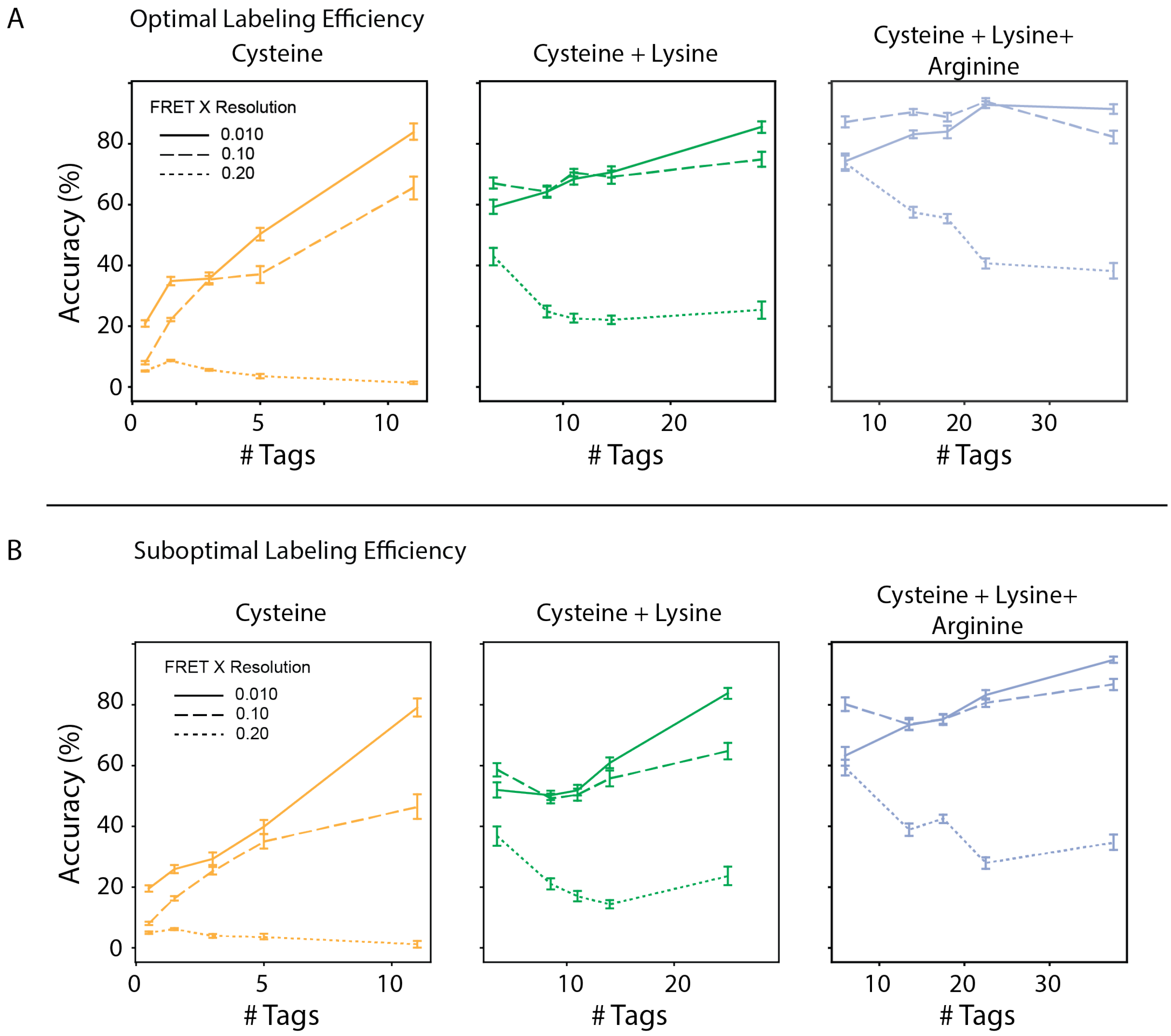

Supplementary figure 6: SVM classifier accuracy on simulated fingerprints for 311 proteins at different resolutions. Related to figure 4C.

Average classifier accuracy versus the number of tagged residues in structures, aggregated in five groups with similar numbers of tags, at different resolutions. Data are shown for **(A)** optimal labeling quality (i.e. 100% efficiency, 100% specificity) and **(B)** suboptimal labeling quality (see supplementary table 3 for efficiency and specificity), for three combinations of tagged residues (C, C + K, and C+ K + R). Whiskers denote two standard deviations.

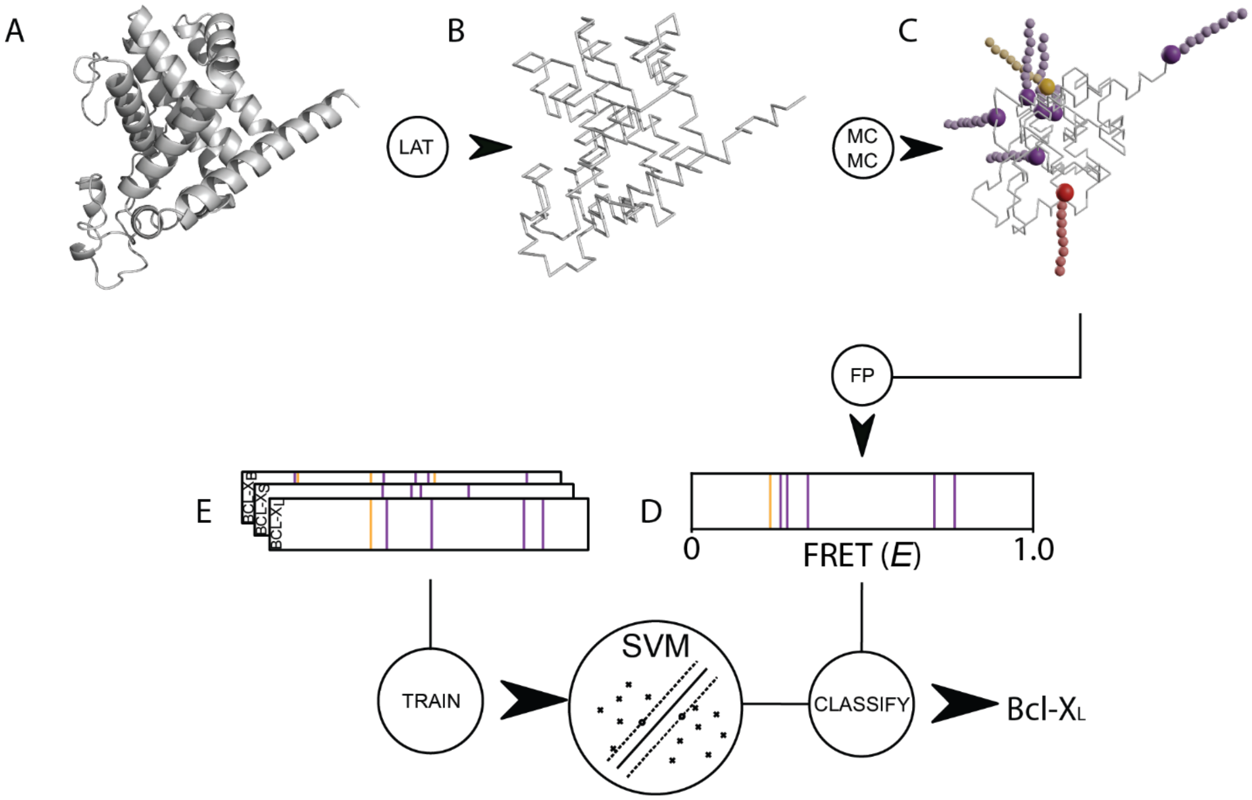

Supplementary figure 7: Schematic of FRET X fingerprinting simulation and classification pipeline used in this work. Related to STAR methods.

(A) Simulation starts from a fully atomistic structure, **(B)** which is first converted into a lattice model. In the lattice model all residues are reduced to their Cα positions. **(C)** Residues to which docking strands must be attached are marked, after which the structure is randomly mutated using a Markov chain Monte Carlo (MCMC) process, until docking strands no longer experience steric hindrance from the rest of the structure. **(D)** The MCMC process then continues while snapshots are taken at regular intervals. Donor-acceptor dye distance for each dye pair is averaged over all snapshots and translated into a FRET efficiency. Combined, the FRET efficiencies form the final fingerprint for this molecule. **(E)** A support vector machine (SVM) is trained on a set of fingerprints with known identities, after which it can be used to classify unseen fingerprints.

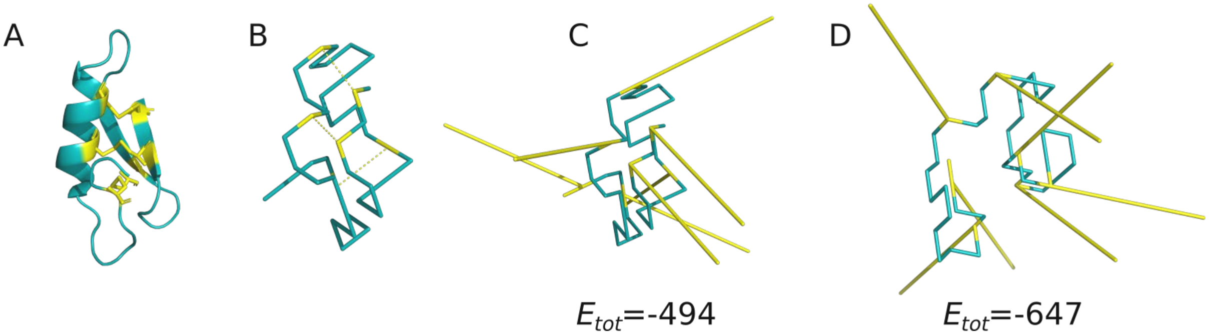

Supplementary figure 8: L-plectasin structure (3E7U) in four steps of the fingerprinting pipeline. Related to STAR methods. **(A)** Fully atomistic structure as recorded in the RCSB protein database (PDB). Six cysteine residues (yellow) form the three disulfide bridges that connect the N-terminal alpha helix and C-terminal beta sheet. **(B)** The structure after translation to a lattice model. Disulfide bridges are marked as dotted lines. **(C)** Cysteines are targeted for tagging. Upon attachment of tags, cysteines lose their ability to interact with residues, thus cysteine bonds are lost. **(D)** After energy minimization, structure has largely been lost. Figures generated in pymol v2.3.0.

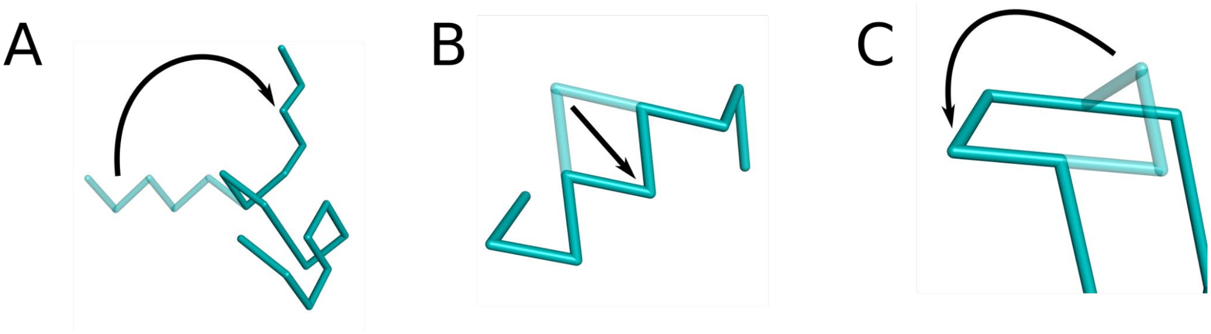

Supplementary figure 9: The three structural modifications applied during energy minimization of lattice models. Related to STAR methods.

(A) A branch rotation rotates all pseudo-atoms before or after a chosen pseudo-atom, **(B)** a corner flip changes the position of a single pseudo-atom to another vertex connected to the neighboring pseudo-atoms and **(C)** a crankshaft move does the same for two neighboring pseudo-atoms simultaneously. Figures generated in pymol v2.3.0.

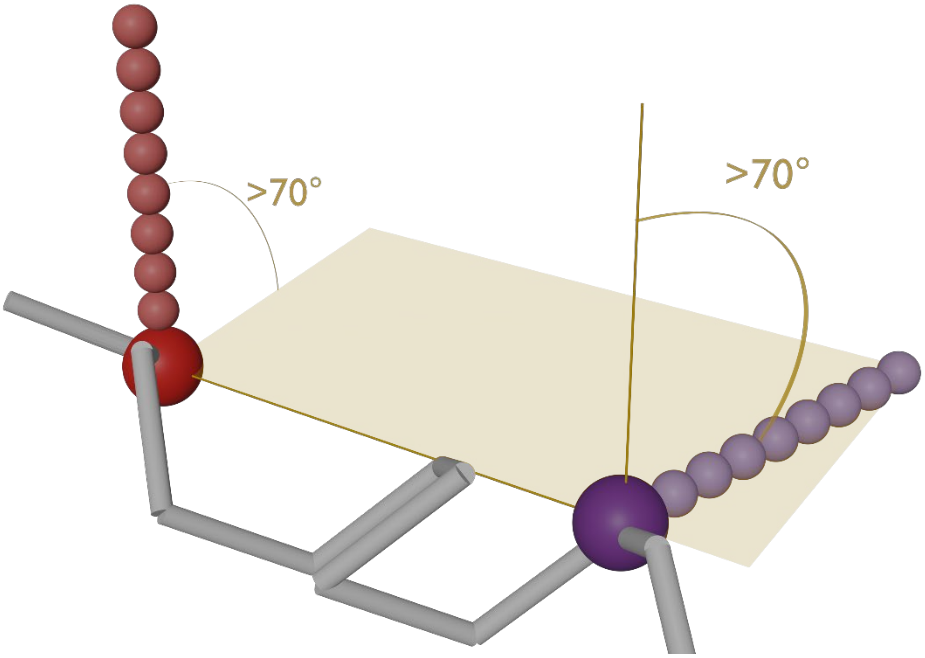

Supplementary figure 10: Illustration of the tag repulsion implementation of the lattice model. Related to STAR methods. If the distance between two tagged pseudo-atoms is found to be less than 20Å, both the angle and the dihedral angle should be larger than 70° to obtain a valid tag position. Figure was generated in Blender 2.93.0.

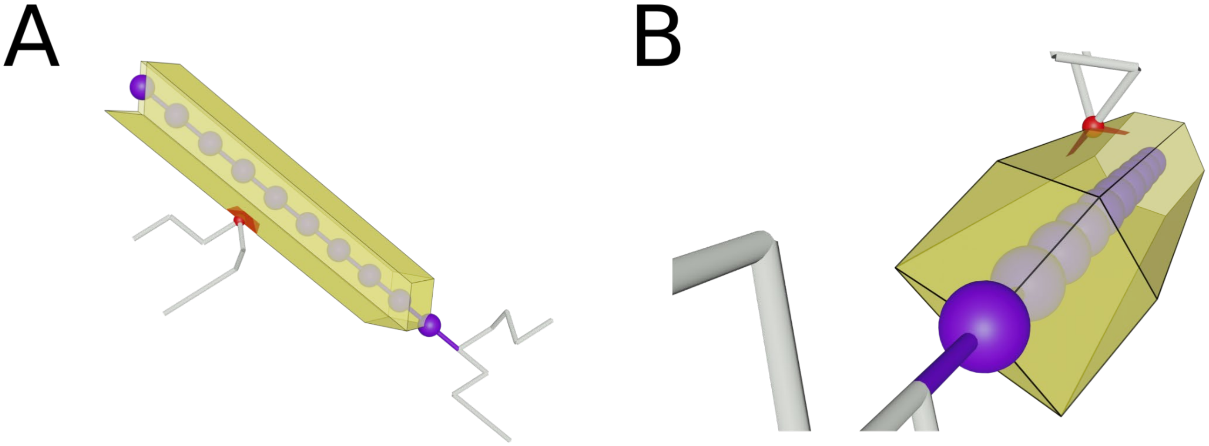

Supplementary figure 11: Lattice space occupied by a DNA-tag. Related to STAR methods.
Shown are a (A) side and (B) bottom view of an on-lattice backbone of the DNA-tag (purple), and the space it is assumed to occupy to account for the additional bulkiness of DNA nucleotides, when compared to amino acids. In this example, a single pseudo-atom from a different part of the structure (red) is incurring steric hindrance. Figure was generated in Blender 2.93.0.

Supplementary table 1: Single-molecule peptide constructs. Related to STAR methods.

Peptide	Sequence (N to C)	Modification	Supplier
K1C10	KAGERDNFACHMALVPVAAN DENYALAAAANDENYALAAA	Biotin-Ahx N-terminus	Biomatik (CAN)
K1C20	KAGERDNFAPHMALVPVAAC DENYALAAAANDENYALAAA	Biotin-Ahx N-terminus	Biomatik (CAN)
K1C30	KAGERDNFAPHMALVPVAAN DENYALAAACNDENYALAAA	Biotin-Ahx N-terminus	Biomatik (CAN)
K1C40	KAGERDNFAPHMALVPVAAN DENYALAAAANDENYALAAC	Biotin-Ahx N-terminus	Biomatik (CAN)

Supplementary table 2: Single-molecule DNA constructs. Related to STAR methods.

DNA Strand	Sequence (5' - 3')	Modification	Supplier
Donor imager strand	AGATGTAT	3' Cy3	Ella Biotech (GmbH)
Acceptor imager strand	AATGAAGA	3' Cy5	Ella Biotech (GmbH)
Donor docking sequence	TATACATCTAT	5' Maleimide	Biomers.net (GmbH)
Acceptor docking sequence	TTCTTCATTACT	5' Azidobenzoate	Biomers.net (GmbH)

Supplementary table 3: Labeling probabilities under suboptimal conditions. Related to STAR methods.

Chemistry	Target	P(target labeling)	Off-target residue	P(Off-target labeling)	Reference
Maleimide thiol reaction	C	90%	K	1%	Boutureira et al. [31]
NHS ester-mediated derivatization	K	90%	S,Y,T	1%	Abello et al.[32]
Arginine derivatization	R	90%	Any	0.5%	Thompson et al. [33]
